# Supplementary material for: Adaptation to hand-tapping affects sensory processing of numerosity directly: evidence from reaction times and confidence
Source: Proc Biol Sci. 2020 May 27;287(1927):20200801. doi: 10.1098/rspb.2020.0801 (PMC7287367; doi:10.1098/rspb.2020.0801)
Supplement: Data from individual participants [file rspb20200801supp1.pdf]

Supplementary Material to: Adaptation to hand-tapping affects sensory processing of numerosity directly: evidence from reaction times and confidence

Paula A. Maldonado Moscoso[1], Guido M. Cicchini[2], Roberto Arrighi[1] &

David C. Burr[1,2*]

1. Department of Neuroscience, Psychology, Pharmacology and Child Health, University of Florence, Florence, Italy
2. Institute of Neuroscience, National Research Council, Pisa, Italy

DOI: 10.1098/rspb.2020.0801

Visual condition

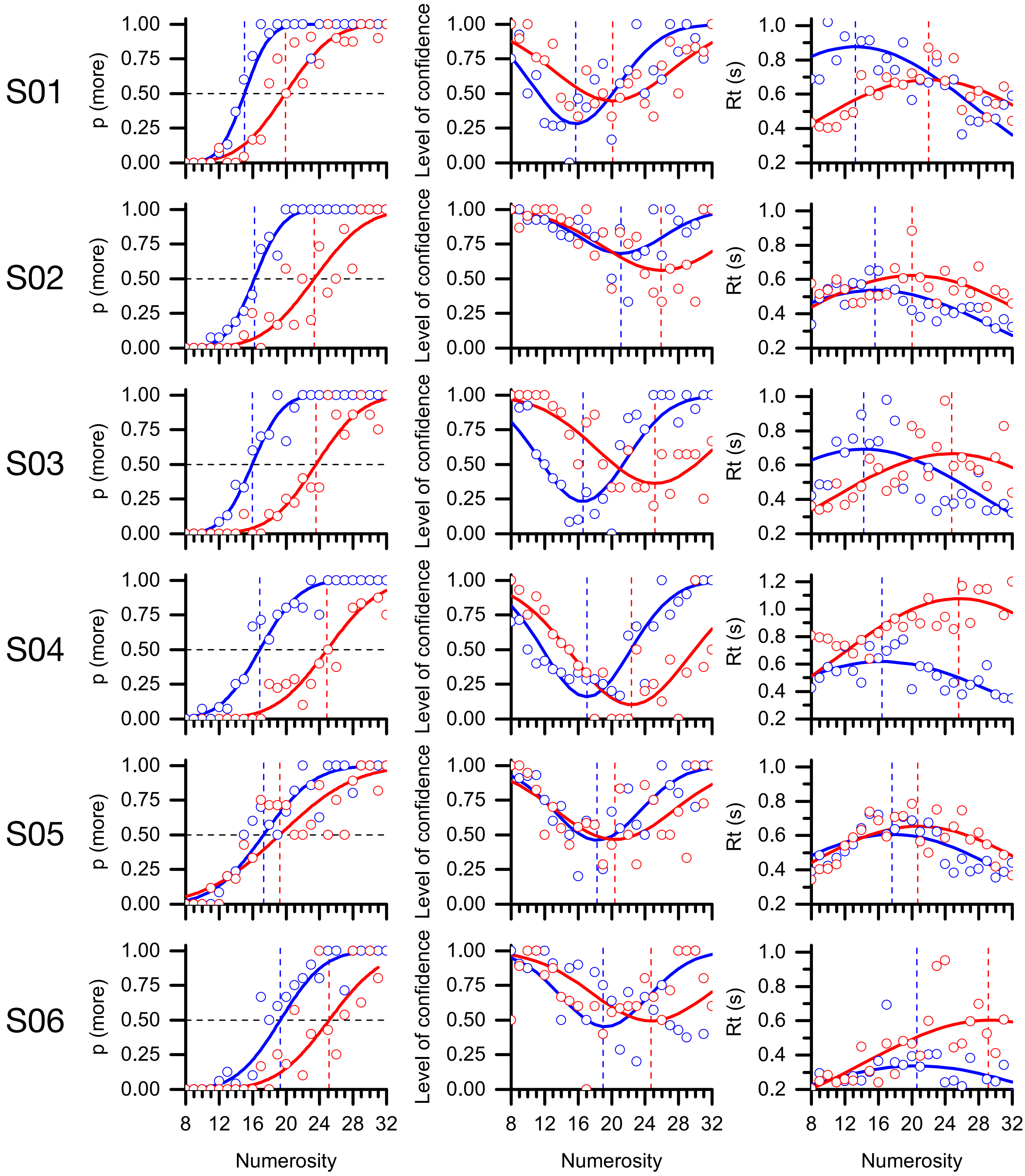

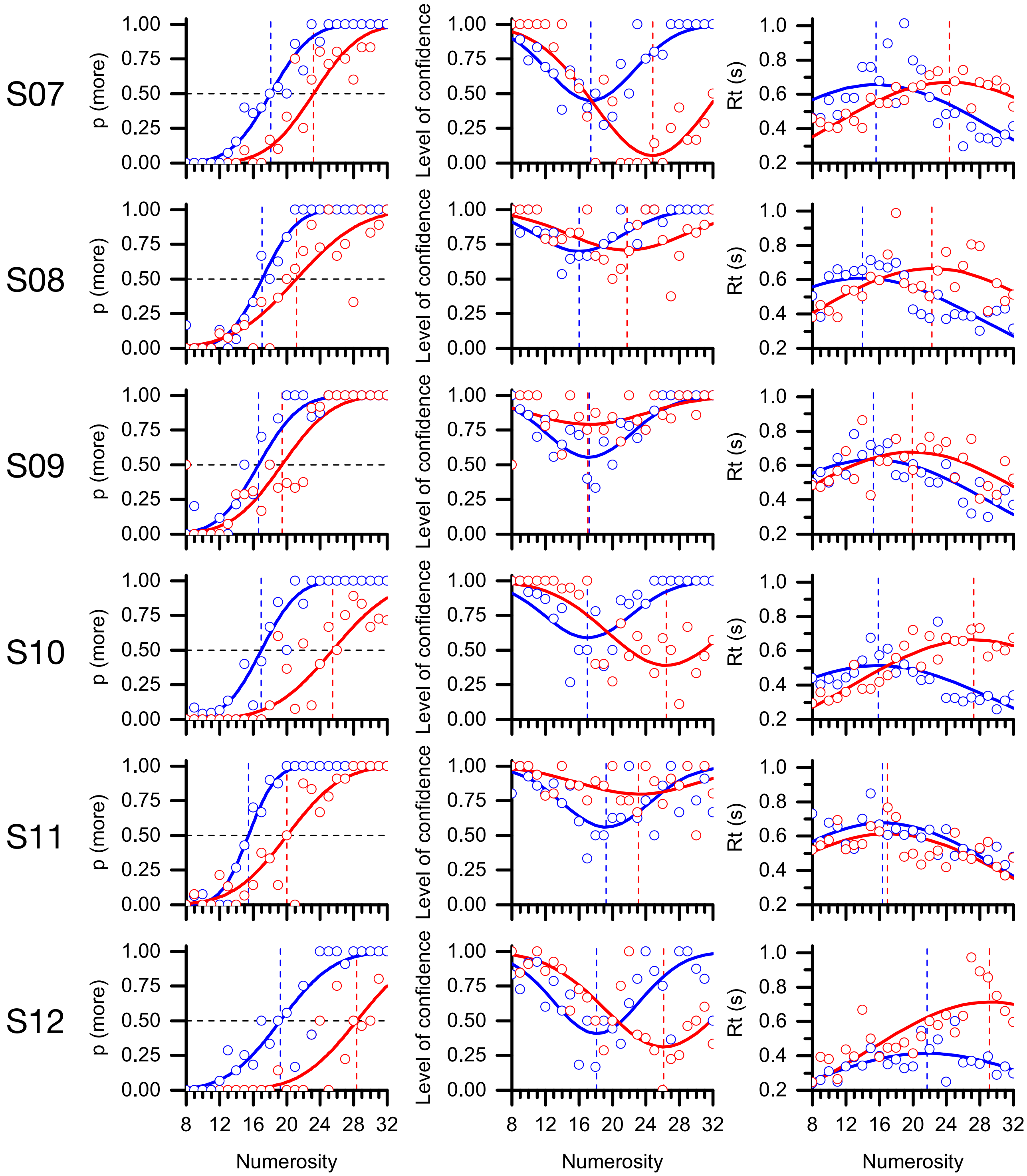

Figure S1. Single-participant plots for the visual condition. The first column plots psychophysical functions of proportion of trials when the test was seen as more numerous than the neutral probe, as a function of physical numerosity (baseline in blue and adapt to high in red). The second column shows average confidence, the third mean reaction-times as a function of physical numerosity. Each row represents a participant. The dashed lines show the PSEs or the peaks of the best-fit gaussians to the confidence or reaction time distributions.

Motor condition

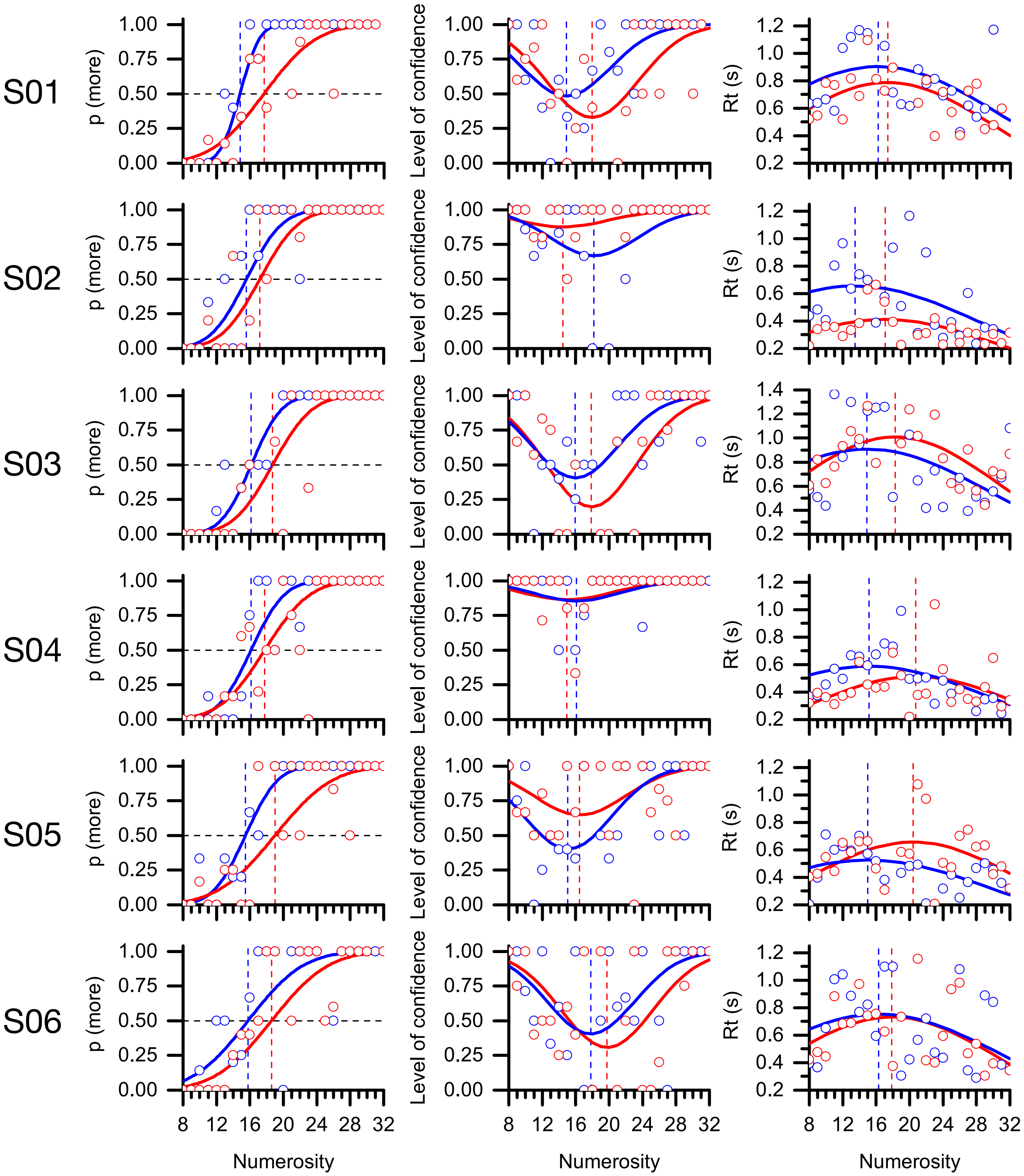

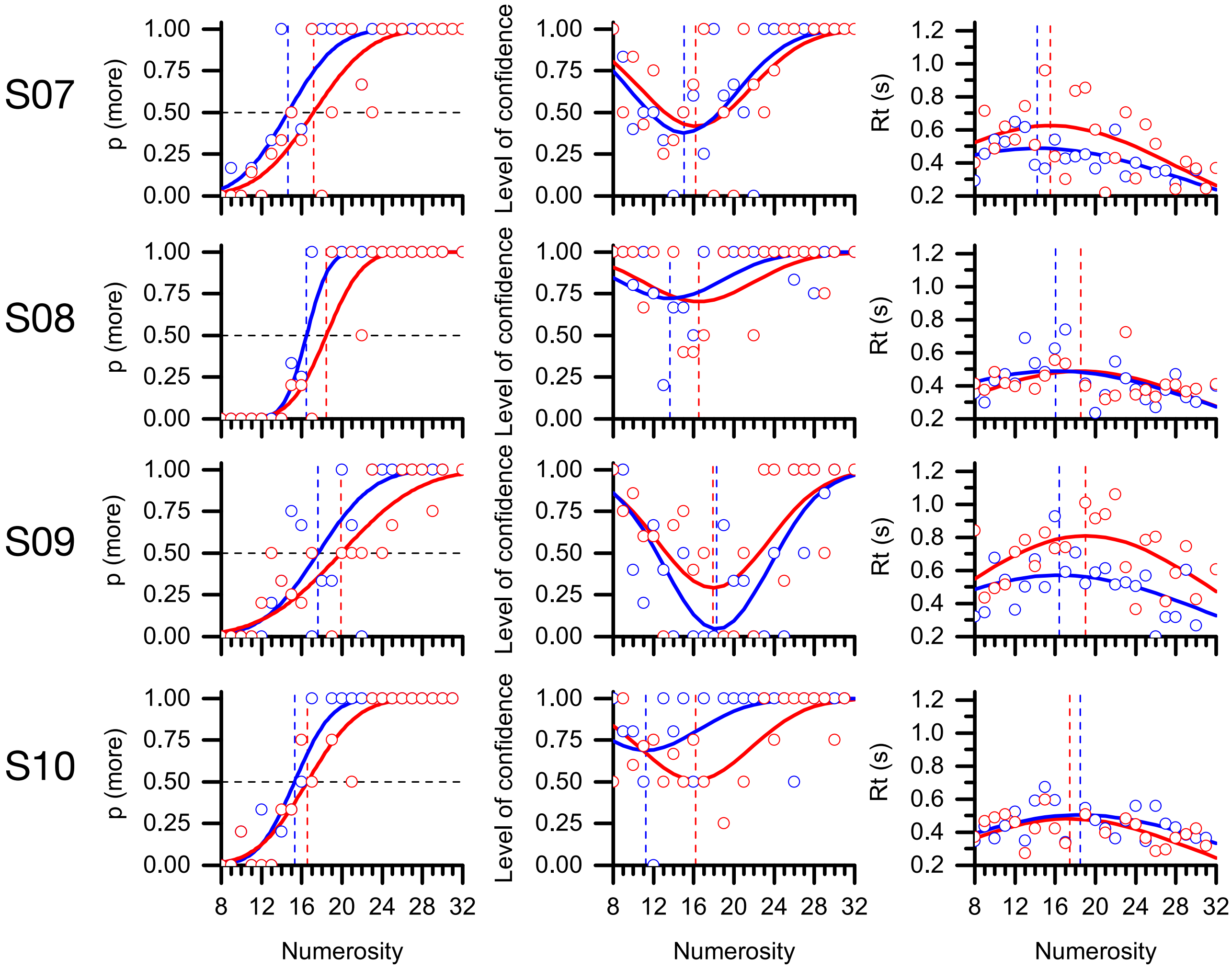

Figure S2. Single-participant plots for the motor condition. Conventions as for Figure S1.

Control condition

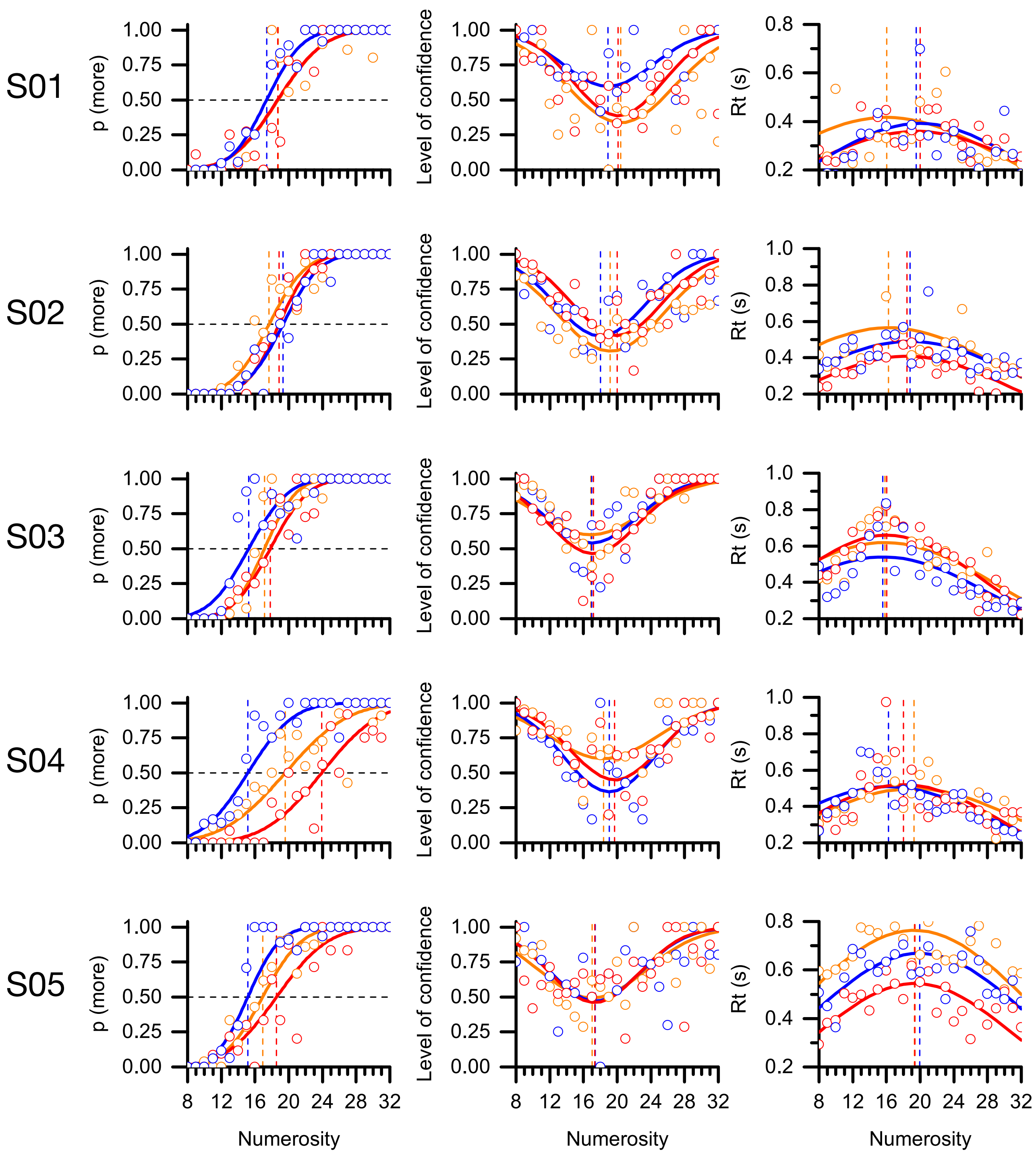

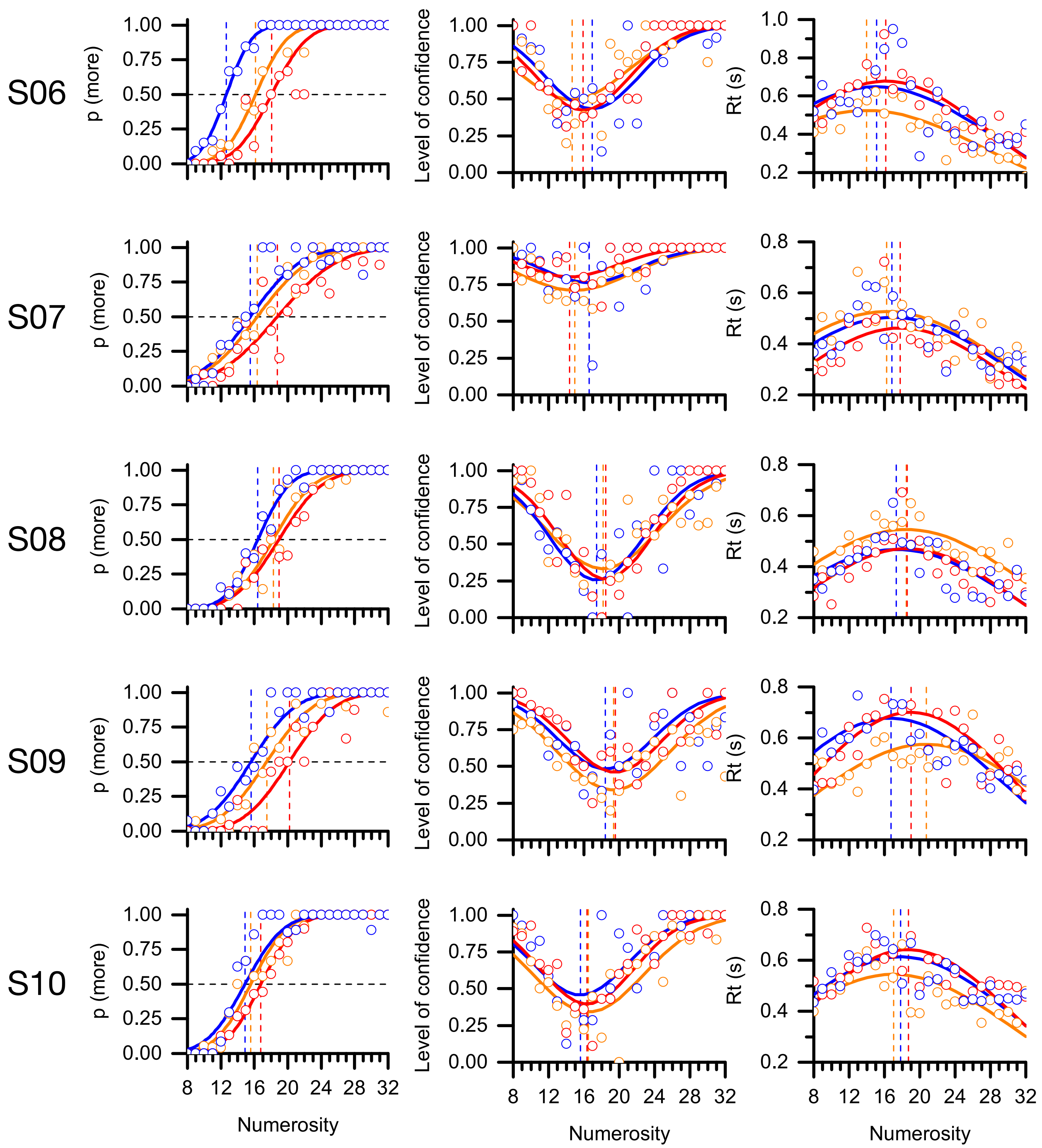

Figure S3. Single-participant plots for the control condition. Conventions as for Figure S1, except that here orange refers to baseline, blue to the "reward-low" condition and red to the "reward-high" condition.

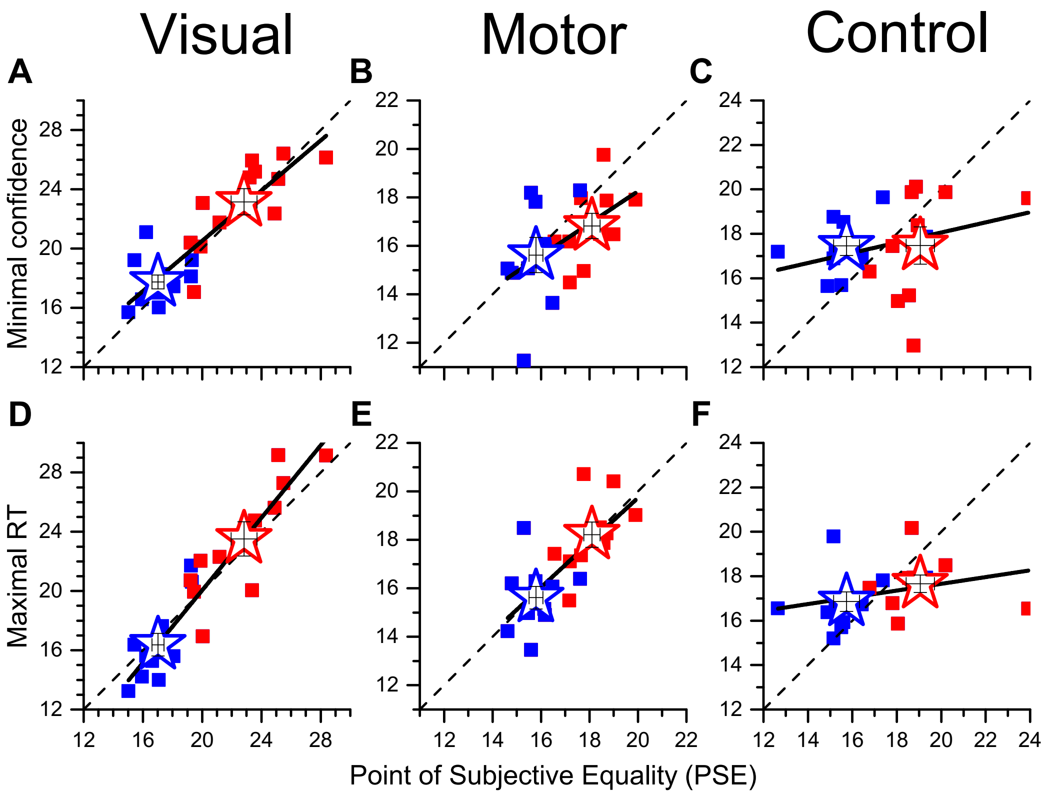

Figure S4. Minima of confidence distributions (A, B, C) and maxima of reaction-time distributions (D, E, F) plotted against PSEs for the visual (A-D), motor (B-E) and control condition (C-F). Single subject data are shown as filled squares and group averages as large open stars. Black lines represent best linear regression lines. Slopes are close to unity in all the adaptation conditions (0.85 and 1.22 for confidence and reaction time for visual adaptation (A-D); 0.66 and 0.92 for confidence and reaction time for motor adaptation (B-E)). Slopes in the control condition are close to zero (0.23 and 0.15 for confidence and reaction time (C-F)).

Conditions			Aggregate		Individual Participants	
			Mean	sem	Mean	sem
Visual	Baseline	PSE	17.05	0.14	17.02	0.39
		Peak Conf.	17.42	0.18	17.53	0.54
		Peak RT	16.03	0.19	16.29	0.79
	Adapt to high	PSE	22.76	0.20	22.82	0.87
		Peak Conf.	23.63	0.34	23.16	0.88
		Peak RT	24.22	0.49	23.35	1.09
Tapping	Slow adaptation	PSE	15.89	0.25	15.79	0.29
		Peak Conf.	15.92	0.44	15.62	0.72
		Peak RT	15.58	0.27	15.64	0.47
	Fast adaptation	PSE	18.15	0.32	18.1	0.34
		Peak Conf.	17.67	0.40	16.82	0.52
		Peak RT	18.15	0.50	18.22	0.52
Control	Baseline	PSE	17.48	0.15	17.39	0.41
		Peak Conf.	17.07	0.29	17.57	0.74
		Peak RT	17.35	0.29	17.59	0.74
	Reward-low	PSE	15.776	0.15	15.734	0.56
		Peak Conf.	17.44	0.32	17.45	0.43
		Peak RT	16.30	0.20	16.86	0.44
	Reward-high	PSE	19.16	0.17	19,06	0.64
		Peak Conf.	17.98	0.22	17.47	0.84
		Peak RT	17.57	0.26	17,67	0.39

Table S1. PSEs, minimal confidence and peak reaction times for all three experiments. Data from the aggregate participant are shown on the middle column, average of individual participants on the right.
